# Learning from the Community to Predict Nutrition Status of Children Aged 6–24 Months in Gulu District, Northern Uganda: A Case Control Study

**DOI:** 10.3390/life12050664

**Published:** 2022-04-29

**Authors:** Muzafaru Ssenyondo, Hanifa Bachou, Richard Bukenya, Richard Kajjura, David Guwatudde

**Affiliations:** 1 Department of Epidemiology and Biostatistics, College of Health Sciences, School of Public Health, Makerere University, Kampala P.O. Box 7072, Uganda; dguwatudde@musph.ac.ug; 2 Department of Community Health and Behavioral Sciences, College of Health Sciences, School of Public Health, Makerere University, Kampala P.O. Box 7072, Uganda; hbachou@fhi360.org (H.B.); rbukenya2021@gmail.com (R.B.); rkajura@musph.ac.ug (R.K.)

**Keywords:** nutrition status, community, caregivers, case and control

## Abstract

The feeding and caring practices of infants and young children are critical to children’s nutrition status and development milestones. Most nutrition studies have focused on unfavorable factors that contribute to malnutrition rather than favorable factors that promote good nutrition status among children. This study aimed at identifying predictors of normal nutrition status among children aged 6–24 months in Gulu District, Northern Uganda. A matched case-control study was conducted on a sample of 300 (i.e., 100 cases and 200 controls) purposively selected children during October–December 2021. Controls were children that had normal nutrition status, whereas cases with undernourished children had at least one type of undernutrition. Logistic regression was used to determine the predictors of good nutrition status using odds ratios (ORs). The mean age of the cases and controls was 15 months (SD ± 6) and 13 months (SD ± 5), respectively. At multivariable analysis, breastfeeding in the first hour of the child’s life (AOR = 3.31 95% CI. 1.52–7.23), use of family planning (AOR = 2.21 95% CI. 1.25–3.90), number of under-fives in the household (AOR = 0.31 95% CI. 0.13–0.73) and hand washing with soap (AOR = 3.63 95% CI. 1.76–7.49) were significantly independently associated with a child’s good nutrition status. Interventions that can improve children’s nutrition status include breastfeeding in the first hour of child’s life, use of family planning methods, child spacing and hand washing with soap.

## 1. Introduction

Globally, 44 percent of infants 0–6 months old are exclusively breastfed, and few children receive nutritionally adequate and safe complementary foods; for instance, in many countries, less than a fourth of infants 6–23 months of age meet the criteria of dietary diversity and feeding frequency that are appropriate for their age [1].

Undernutrition (stunting at 30 percent and wasting at 7.3 percent) accounts for at least half of all the deaths annually in children under five years of age in sub-Saharan Africa [2]. The impact of limited community involvement and poor feeding practices has further increased the magnitude of mortality (5.4 deaths per 1000 live births) among children despite the country reporting sufficient food production throughout the year [3].

Results from the previous Uganda Demographic Health Surveys (2011–2016) indicated that seven percent of all infants were born with low birth weight (weighing less than 2500 g), 29 percent were stunted (short for their age), 11 percent were underweight (small for their age) and four percent were wasted (small for their height) [4].

Several nutrition initiatives have been implemented in Uganda to improve the nutritional status outcomes for children and mothers at both health facility and community levels. These initiatives include promotion of breastfeeding; nutrition education and counselling on maternal, infant and young child feeding; growth monitoring and promotion; integrated management of acute malnutrition; micronutrient supplementation; promotion of consumption of fortified foods; and promotion of household diet diversity [5]. In addition, communication and advocacy initiatives have also been employed for example the Uganda Nutrition Advocacy and communication strategy to promote awareness on nutrition in Uganda.

Despite these initiatives, Infant and Young Child Feeding (IYCF) among children below 24 months is still suboptimal in the country. About 66 percent of children under the age of 6 months are exclusively breastfed, and six in seven children aged 6–23 months, (about 85 percent) do not consume a minimally adequate diet; that is, they do not consume food from at least three food groups at least twice a day [4]. 

In this sub region, only three percent of children aged 6–23 months living with their mother are fed a minimum acceptable diet; seven percent are fed according to minimum dietary diversity (they were fed from at least four food groups); and 25 percent are fed according to minimum meal frequency; that is, they are fed two to four times per day depending on age and breastfeeding status [4]. About 66 percent of children under the age of 6 months are exclusively breastfed.

The Uganda Demographic and Health Survey (UDHS) of 2016 reported that three in ten children aged 6–24 months in the Acholi sub-region are stunted, while four percent are wasted and overweight. These statistics are higher than the national average. Despite the high levels of undernutrition in Acholi sub-region, Gulu district has shown better nutrition outcomes compared with other districts in the same region, hence the need to identify the unique child-feeding and caring practices that may be contributing to better nutrition outcomes of children aged 6–24 months in Gulu district [6]. This study therefore aimed at identifying predictors of good nutrition status among children aged 6 to 24 months in Gulu district, Northern Uganda.

## 2. Materials and Methods

The study was carried out in Gulu district, Acholi sub region in Northern Uganda during October–December 2021. Gulu District is located in Northern Uganda between longitudes 30–32 degrees east and latitudes 02–4 degrees north. There are 70 parishes (54 parishes in the rural sub-counties and 16 wards/parishes in the divisions) and 294 villages. According to the Integrated Food Security Phase Classification report of 2017 [7], 26 percent of the total population in Acholi region was facing stressed food insecurity (IPC Phase 2). Gulu district was selected because it has the highest population level in the Acholi sub-region. All the villages identified to have a malnourished child from the clinics were visited and included in the study. The district has a projected population of 12,730 children below two years, of which 369 of these children are moderately malnourished and receive supplementary feeds. These were excluded from the study. 

A matched case-control study design (matched for age) was used to explore the successful adaptive behaviors and practices of mothers and caregivers with well-nourished children as compared to those with malnourished children in the community. Cases were undernourished children aged 6–24 months (having at least one of the forms of undernutrition, including: underweight (WAZ) and/or stunted (HAZ) and/or wasted (WHZ-scores <−2 Z-scores). Controls were children of the same age group with normal nutrition status (WAZ and HAZ and WHZ-scores ≥−2SD). The study population were children aged 6–24 months, and their mothers/caregivers were the source of information regarding feeding, caring and health-seeking practices for their children. These were the most suitable people to provide information regarding feeding and caring practices of their children since they spend the most time with these children.

A purposively selected sample of 300 children aged 6–24 months (100 as cases and 200 as controls) was included in this study conducted from October to December 2021, considering the following expected breastfeeding rate for Uganda (Po) as a key exposure factor = 43.2% [4]. Breastfeeding was selected because it is the commonest practice (essential nutrition action) recommended for children below 2 years of age [1]; smallest odds ratio expected (R = 2.0), power of 80%. To increase power of the study, 2 controls for each case were enrolled into the study (ratio of controls to cases, c = 2) and a 95% level of confidence was used with Epi Info Software (version 7.0.9.34) based on Fleiss Formula [8].

Cases (stunted, wasted or underweight children) aged 6–24 months were identified from the clinics/health facilities and followed up to their homes. Their corresponding controls (children of normal weight-for-age, normal length-for-age and normal weight-for-length) were identified in the same neighborhood of each case (not more than one village apart). Two controls per each case were identified to increase the power of the study [9]. Nutrition status was determined by calculating Z-scores obtained from computing the weight, length and age of the child (obtained from the child health register). These Z-scores were later graded using the 2006 WHO child growth standards. Cases were randomly selected from health facility registers using simple random sampling. A list of malnourished children attending outpatient nutrition therapeutic care (OTC) in a selected health facility was generated, and each name was assigned unique identification numbers from which the cases were selected at random to be included in the sample. This was done during fixed Outpatient Therapeutic Care (OTC) days on which these cases received treatment. The randomly selected cases where then followed up to their homes in the villages. 

Both the cases and controls shared similar characteristics in the context of residence (same or nearby village) and age group (6–24 months), except for their anthropometric nutrition status. The controls were picked from the same village as the cases, and when this was not possible, controls were then identified from the nearby villages (not more than 1 village away from the case’s village). In case more than two eligible children (cases or controls) were identified in a household, the reference child considered for the study was randomly selected from those identified to be eligible. Both cases and controls were not refugees, had no severe/chronic health problems and were not enrolled in a supplementary feeding program [5].

In this study, the dependent variable was the child’s nutrition status (undernutrition based on Z-scores for weight-for-age, length-for-age and weight-for-length indices). This was a binary outcome with those that are undernourished (cases) with less than −2 SD Z-scores for at least one of the indices (WAZ or WHZ or HAZ) and those that had normal nutrition status (controls) with above or equal to -2 SD Z-score for all the nutrition indices. These Z-score values were calculated by the research assistants from computing the child’s weight, length and age and then comparing these Z-scores with the WHO reference growth charts. Independent variables included social demographic factors, feeding practices, caring practices and health-care-seeking practices. 

A semi-structured interviewer-administered questionnaire was used to collect primary data from participants. The interview questionnaires were pre-tested on 30 participants (as recommended by Perneger et [10]) in areas different from the study sites (neighboring district of Omoro) but with similar characteristics in terms of common food staples, cultural practices and livelihood [4]. For anthropometric measurements, recumbent length was measured using seca-infantometer 416 (length board) and child’s weight measured using the Seca 874 U electronic scale manufactured by gmbh & co.kg located in Humburg-Germany (capacity 150 kg) with a precision of 100 g. Age of the child was obtained from the child’s health cards and birth certificates, and where not available, it was obtained from the mother’s/caretaker’s reported age for the child.

Identification of cases and controls aged 6–24 months was conducted according to the nutritional status parameters: weight-for-age (underweight), length-for-age (stunting) and weight for length (wasting). Measurement values of weight, length and age of these children were entered in Epi-data software version 3.1, cleaned and after exported to ENA for SMART software version 2011, to obtain the children’s Z- scores. The software automatically classified these children as either normal, underweight, stunted or wasted (less than -2SD Z-scores). The proportion of undernourished children was determined, and results were presented in percentages (%).

Quantitative data from the questionnaires were entered into Epi-data software and after exported to STATA version 14 and ENA for SMART software and cleaned before analysis. To identify key child-feeding, caring and health-seeking practices and behaviors of mothers/caregivers associated with the nutrition status of their children, the primary dependent variable of interest was nutritional status, as determined by WAZ or HAZ or WHZ. Each child was classified as having good/optimal nutritional status if their WAZ was ≥−2 standard deviations, if their HAZ was ≥−2 standard deviations and if their WHZ was ≥−2 standard deviations (coded as 1). Otherwise, they were classified as not having good nutritional status (coded as 0). 

Logistic regression was used to determine the independent predictors of good nutrition status while controlling for confounders, using odds ratios (ORs) as the measure of association. A *p*-value less than 0.05 was considered statistically significant at 95% confidence interval. All independent variables identified at bivariable analysis with a conservative level of significance set at *p* < 0.2 were included in the logistic regression model. Multi-collinearity was checked for by conducting a correlation coefficient among independent variables. One of the independent variables was selected among variables whose correlation coefficient was >=0.4 to be included in the model. A stepwise approach was used in building the model. Variables with higher *p*-values were removed from the model and retained the ones with *p*-value of less than 0.05. The chi-square goodness of fit was used to determine the best multivariable analysis model. Some variables that were significant at bivariable analysis (partners support, child immunized, marital status and family size) were removed from the final model due to collinearity and to obtain the best goodness of fit. The final model had a non-significant chi-square *p*-value of 0.359, indicating a non-significant difference between the final model and the standard.

A logistic regression model was constructed to identify independent factors associated with good nutrition status with probability values of <0.05 considered significant. Results were presented in frequencies, percentages, and crude and adjusted ORs. The social variables in the study included: age of the child in completed months, sex of the child (male or female), marital status of the mother/caregiver (married or not married), occupation and place of work (work that one earns from and does on a day-to-day basis), highest level of education (none, primary incomplete, primary complete, O-level incomplete, O-level complete, A-level, higher institutions of learning and university), income earned per day, family size, duration of stay at work (morning, evening, all day, all night). Moreover, participation in community events, such as teachings, sports, SACCOs, advocacy, leadership and trade, was noted, as well as residence (rural or urban). Social economic status was measured based on the family head’s income.

Family size was categorized as less or equal to 5 individuals and more than 5 individuals in a household, based on similar studies conducted in Ethiopia and Pakistan [2,11,12]. Education level was divided into three categories, that is: no formal education, primary, and secondary level or higher, adapted from a similar case-control study in South-Western Uganda [13]. Breastfeeding frequency was categorized as less than 8 times/day or 8 times/day and above, adapted from the MoH guidelines on Maternal Infant Young Child and Adolescent Nutrition—MIYCAN [5], which recommend breastfeeding of infants at least eight times a day.

Family earnings were categorized as less than 1 US dollar/day or above based on the World Bank classification rate of per capita income that classifies poverty as daily income of less than a dollar. ANC visits were categorized as less than four visits or four and above, adapted from the Maternal Nutrition Guidelines of Uganda [5], which recommend at least four antenatal care visits for the pregnant mothers. Mother’s/caretaker’s age was taken as completed years as mentioned by mother/caretaker. It was classified into one of three categories: <19 years, 20–35 years and ≥35 years, adapted from a similar community case-control study conducted in Pakistan [2].

Information regarding feeding practices was obtained using a semi-structured questionnaire, and this included: breast feeding status (exclusive breastfeeding, mixed feeding, replacement feeding or complementary feeding), duration of breastfeeding (below 6 months, before one year and two years and beyond), introduction of complementary foods (before, at or after 6 months), type of feeding (hand, spoon, bottle or cup), foods and drinks fed to the child so far today (listing all food and drink including breast milk), what to do when child refuses to eat, knowledge on proper foods for children, foods to feed the child while sick, snacks given to the child, and appropriate age for cessation of breastfeeding. 

Care practices were obtained using a semi-structured questionnaire that included: ways family members and children interact (psycho-social care) and early childhood stimulation, hand washing with soap, disciplining a child when he/she does wrong, perceptions on the most important needs of the child, playing with the child (when and what games), knowledge on WASH, partner/spouse assistance in caring for the child, and child care during illness. 

Health-seeking practices were obtained using a semi-structured questionnaire that included: preventive health practices (age-appropriate immunization), proper management of child illness, child illness suffered in the last 2 weeks (diarrhea, malaria, pneumonia or other diseases), common child illnesses suffered (managing the situation, when to seek treatment and where, who to consult first), hygienic practices (WASH, including body, food, and environment), use of mosquito nets, and use of child spacing/ family planning. These feeding, caring and health-seeking practices were adapted from the Ministry of Health Policy Guidelines on Infant and Young Child Feeding, IYCF [5].

Ethical approval to conduct the study was obtained from the Higher Degrees Research and Ethics committee of Makerere University, School of Public Health. In addition, an introductory letter was obtained from the Gulu District Health Office (DHO), allowing us to conduct the study based on the health facilities in the selected villages. Mothers/caretakers provided consent to enroll children in this study by either signing or putting their thumb print on the consent forms before participating in the study.

## 3. Results

### 3.1. Characteristics of Study Participants

In this study, a total of 300 children aged 6–24 months (100 cases and 200 age-matched controls) were enrolled. The results regarding the background characteristics are presented in Table 1 below. The mean age of the cases and controls was 15 months (SD ± 6) and 13 months (SD ± 5), respectively. There were 148 boys and 152 girls in the sample as shown in Table 1 below. Of the cases and the controls, 57 (57%) and 91 (45.5%) were males, respectively. Marital status and age of caretaker, family size, number of children under-five in the house and family earnings per day were the characteristics that significantly differed among the cases and controls.

A total of 275 (91.7%) of caretakers were mothers of these children. The majority (201 (67.0%)) of the caretakers reported to have attained at least the primary level of education, with 64 (21.3%) having secondary education and above, and 35 (11.7%) having no formal education. Regarding the marital status of the caregiver, 39 (39.0%) of the caregivers of cases were in polygamous marriage compared to 42 (21.0%) of caregivers of controls. The majority (87%) of households had less than three children below five years of age, with 76 (76.0%) and 185 (92.5%) among cases and controls, respectively.

The average household size was 6 (SD ± 2) and 5 (SD ± 2) individuals among the cases and the controls, respectively. Farming was the major source of income for both groups (79% of cases and 75% controls, respectively). The majority of the households 260 (86.7%) earned less than a dollar in a day. Regarding participation in community events, 51 (51.0 %) and 119 (59.5%) of cases and controls engaged in SACCOs, respectively (Table 1).

Fifty-seven percent (57%) of the boys were undernourished compared to 43% of the girls. However, the difference was not statistically significant (*p* = 0.06), as shown in Table 1 above.

### 3.2. Comparison of Child-Feeding, Caring and Health-Seeking Practices between Caretakers of Cases and Controls

#### 3.2.1. Child-Feeding Practices of Caretakers

The results presented in Table 2 below show the child-feeding practices. The majority (294 (98.0%)) of the cases and controls were ever breastfed (97.0% and 98.5% of cases and controls, respectively). Breastfeeding was initiated within the first hour of birth in 77 (79.4%) of the cases and 177 (89.9%) of the controls, and the difference between the two groups was significant (*p* = 0.014). Complementary feeding was started before six months in 90 (45.0%) of the controls and in 57 (57.0%) of the cases. A total of 130 (66.0%) controls were breastfed more than eight times a day compared to 44 (45.4%) cases, and the difference was significant (*p* = 0.001). Feeding a child using hands was practiced in 181 (90.5%) of the controls and 94 (94.0%) of the cases. Eighty-four (42.0%) and 40 (40.0%) of the controls and the cases, respectively, reported feeding their children more frequently when they were sick.

#### 3.2.2. Caring Practices of Caretakers

As shown in Table 3 below, more caretakers for the controls used soap for hand washing 180 (90.0%) than the cases (73 (73.0%), and the difference between the two groups was statistically significant (*p* < 0.001). A total of 130 (65.0%) and 59 (59.0%) caretakers of controls and cases, respectively, were educated on water, sanitation and hygiene (WASH). Regarding disciplining a child, 117 (58.5%) and 57 (57.0%) caretakers of controls and cases (respectively) talked to the child when she/he had done wrong. In both groups, the majority of caretakers (82% in controls and 76% in cases) reported food to be the most important basic need of a child. Regarding partner support, 166 (83.0%) and 70 (70.0%) caretakers of controls and cases (respectively) reported that ensuring food availability in the household was the common assistance received from their spouse, and the difference was significant with *p* = 0.01.

#### 3.2.3. Health-Seeking Practices

The data presented in Table 4 below indicate that more mothers of controls (92.5%) attended antenatal care (ANC) services than mothers of cases (82.0%), and this difference was statistically significant (*p* = 0.006). Eighty four percent (84.0%) of mothers of controls had more than four ANC visits compared to mothers of cases (65.0%) during pregnancy, and the difference was significant (*p* < 0.001). The majority of the cases and controls were immunized—94 (94.0%) and 197 (98.5%), respectively—and the difference between the two groups was statistically significant (*p* = 0.031). Fever/malaria was the commonest illness suffered by children, with 162 (81.4%) of controls and 77 (77.0%) of cases reported to suffer from malaria fever most times. Other illnesses reported were measles, diarrhea, ARIs and skin and eye diseases.

Regarding management of a sick child, 178 (89.0%) caretakers of controls and 80 (80.0%) of those of cases took their children to a health facility when they fell sick. More of the cases (84 (84.0%)) suffered an illness in the past two weeks compared to the controls (134 (67.0%)), and the difference was statistically significant (*p* = 0.002). Mothers of controls had a smaller number of pregnancies compared to mothers of cases; 150 (75.0%) mothers of controls had conceived less than four times compared to 52 (52.0%) mothers of cases, and the difference was statistically significant (*p* < 0.001). In addition, more of the controls (80.5%) slept under a Long Last Insecticide Net (LLIN) than cases (68.0%) the previous day, and the difference between the two groups was statistically significant (*p* = 0.016).

### 3.3. Feeding, Caring and Health-Seeking Practices of Mothers/Caregivers Associated with Optimal Nutrition Status of the Children

At bivariable analysis (as shown in Table 5 below), early initiation of breastfeeding, breastfeeding a child more than eight times a day, hand washing with soap, ANC attendance, having no illness in the last two weeks, use of family planning methods and sleeping under a mosquito net were practices significantly associated with child’s optimal nutrition status. The child being ever breastfed was not significantly associated with good nutrition status. The odds of being well nourished among children who were initiated on breastmilk in the first hour life were 2.3 times the odds among children who were initiated on breastfeeding after an hour, and it was statistically significant (COR = 2.29, 95% CI. 1.16–4.49). The odds of being well nourished among children who were breastfed at least eight times/day were 2.3 times the odds among children breastfed less than eight times a day, and it was statistically significant (COR = 2.34, 95% CI. 1.42–3.84).

The odds of a child being well nourished among caretakers who used soap for hand washing was 3.3 times the odds among caretakers who did not use soap, and it was statistically significant (COR = 3.34, 95% CI. 1.78–6.31). Mothers who attended antenatal care services (ANC) during pregnancy were 2.71 times more likely to have well-nourished children than mothers who did not attend ANC during pregnancy, and it was statistically significant (COR = 2.71, 95% CI. 1.30–5.63). The odds of having a well-nourished child among mothers who attended at least four ANC visits were 2.83 times the odds among mothers who had less than four ANC attendances, and it was statistically significant (COR = 2.83, 95% CI. 1.33–4.45). Children who had no illness in the past two weeks were significantly more likely to be well nourished (COR = 2.59, 95% CI. 1.40–4.76). 

The odds of having a well-nourished child among mothers who were using any family planning method were 2 times the odds for mothers who were not using any family planning method, and the association was statistically significant (COR = 2.04, 95% CI. 1.24–3.34). Mothers who had conceived more than three times were 64% less likely to have well-nourished children than mothers who conceived three or less times, and it was statistically significant (COR = 0.36, 95% CI. 0.22–0.60). Children who slept under a Long Last Insecticide Net (LLIN) the previous day were 94% more likely to be well nourished than children who did not sleep under a mosquito net, and the association was statistically significant (COR = 1.94, 95% CI. 1.12–3.36).

### 3.4. Social Factors Associated with Optimal Nutrition Status of Children

As shown in Table 6 below, the education level and marital status of the caretaker, the number of under-fives in the household and the daily family income were statistically significantly associated with the optimal nutrition status of the children.

The odds of having a well-nourished child among caretakers who completed primary and secondary or higher level of education were 2.3 and 2.7 times, respectively, the odds among caretakers who had no formal education, and this was statistically significant (COR = 2.27, 95% CI. 1.09–4.69 and COR = 2.71, 95% CI. 1.15–6.38).

Caretakers in a polygamous marriage were 72% less likely to have well-nourished children than caretakers who were in a monogamous marriage, and this was statistically significant (COR = 0.28, 95% CI. 0.15–0.53). Cohabiting and not being married were not statistically significantly associated with optimal child anthropometric nutrition status.

Households with more than two children aged below five years were 61% less likely to have well-nourished children than households with two or less under-fives, and this was statistically significant (COR = 0.39, 95% CI. 0.19–0.82). In addition, the odds of having a well-nourished child among families earning more than 1 USD/day was 2.93 times the odds among families earning 1 USD or less/day, and this was statistically significant (COR = 2.93, 95% CI. 1.18–7.27).

### 3.5. Multivariable Analysis

At multivariable analysis, breastfeeding in the first hour of child’s life, use of family planning methods, smaller number of children under-five in a household and hand washing with soap were significantly independently associated with the child’s optimal anthropometric nutrition status (Table 7). However, frequency of breastfeeding, ANC attendance, sleeping under a mosquito net, education level of caretaker, marital status and family earnings per day did not show a statistically significant relationship with the child’s normal nutrition status at multivariable analysis.

Table 7 below shows that the odds of being well nourished among children who were initiated on breastmilk in the first hour life were 3.3 times the odds among children who were initiated on breastfeeding after an hour, and the difference was statistically significant (AOR = 3.31, 95% CI. 1.52–7.23). Mothers who used any family planning method were 2.21 times more likely to have well-nourished children compared to mothers who were not using any family planning method, and this difference between groups was statistically significant (AOR = 2.21, 95% CI. 1.25–3.90). Furthermore, households with more than two children aged below five years were 69% less likely to have well-nourished children than households with two or less children under-five, and this difference between the two groups was statistically significant (AOR = 0.31, 95% CI. 0.13–0.73). The odds of a child being well nourished among caretakers who used soap for hand washing was 3.63 times the odds among caretakers who did not use soap for hand washing, and the difference was statistically significant (AOR = 3.63, 95% CI. 1.76–7.49).

## 4. Discussion

This study was carried out to identify practices of mothers/caregivers associated with optimal nutrition status of their children aged 6–24 months in Gulu district, Northern Uganda. In this study, breastfeeding in the first hour of the child’s life, child spacing through use of family planning methods, having a smaller number of children under five in the same household, having no history of illness in the last 2 weeks, and hand washing with soap were practices significantly and independently associated with a child’s good nutrition status.

On comparison of child-feeding, caring and health-seeking practices of caretakers of cases and control groups, the following practices were significantly varying between the two groups: initiation of breastfeeding, times breastfed since the previous day, hand washing with soap, illness suffered in the last two weeks and use of family planning methods.

The results show that breastfeeding was initiated within the first hour of the child’s birth in 79.4% of the cases and 89.9% of the controls, and the difference was significant (*p* = 0.014) between the two groups. A similar finding from a Positive Deviance/Hearth study in five communities of rural Kenya showed a significant difference in breastfeeding behaviors between mothers of controls and cases (*p* ˂ 0.023), with 60.4% of the caregivers/mothers of well-nourished children feeding their infants within an hour compared to 33% of the caregivers of malnourished children [14]. Commencement of breastfeeding immediately after birth ensures that the newborn receives the “first milk” (colostrum), the baby’s first immunization from diseases that impacts the child’s nutrition status [15].

In addition, the proportion of children breastfed more than eight times a day was significantly higher among the controls than the cases (*p* = 0.001). This finding is consistent with a positive deviance study among sixty slum children conducted in India [16], where 86% of the well-nourished children were breastfed at least eight to nine times per day compared to 20% of the cases. The results show compliance with the Uganda Maternal Infant, Young Child and Adolescent Nutrition (MIYCAN) guidelines that recommend infants to be breastfed at least eight times in a day [17].

Furthermore, the percentage of mothers/caretakers using any family planning methods among the controls was statistically significantly different from that of the cases (*p* = 0.004). This finding suggests that family planning as a means of child spacing plays a key role in the child’s nutrition status. This is supported by study findings from Ethiopia which reported that family planning was positively associated (AOR = 2.54; 95% CI. 1.12–5.77) with well-nourished children [18]. These results clearly indicate that well-spaced children are given adequate attention by their mothers/caretakers to promote their growth and nourishment [13,19]. Overall, the family should have children they are able to provide with enough resources, of which food is key.

In addition, a higher percentage of mothers/caretakers for the controls used soap for hand washing (90.0%) than the cases (73.0%), and the difference between the two groups was statistically significant (*p* < 0.001). A similar finding in India [20], also reported that use of a hand washing agent (soap/ash/mud) compared with water alone was strongly and positively associated with normal nutrition status (height for age) (*p*  =  0.001). The 2013 UNICEF report on “Improving child nutrition: The achievable imperative for global progress” states that proper hand washing with soap can prevent nearly half of all cases of childhood diarrhea which in turn reduces the risk of malnutrition [21]. The UNICEF 2013 report further estimated that drinking clean water and hand washing with soap can prevent the loss of nutrients through diarrhea and reduce stunting by up to 15% in children under the age of five, giving them a better chance of maintaining good nutrition and growing up to thrive [21].

At multivariable analysis, early initiation of breastfeeding, having no history of illness in the last two weeks, hand washing with soap and use of family planning were practices significantly associated with optimal nutrition status. Mothers who initiated breastfeeding in the first hour of a child’s life were more likely to have well-nourished children than mothers who initiated breastfeeding after an hour. This result is consistent with the World Health Organization’s recommendation of initiating all newborns on breastfeeding within the first hour of their life [15]. Evidence drawn from a meta-analysis from over 63 developing countries shows that early initiation of breastfeeding prevents newborn infections; averts newborn death due to sepsis, pneumonia, diarrhea and hypothermia; and facilitates sustained breastfeeding [22].

In addition, a cross-sectional study conducted in Kenya observed that children who were breastfed after one hour of birth were twice as likely to be stunted as compared to those who breastfed within one hour of birth [23]. In Uganda, only 66% of newborns are breastfed within the first hour of birth [4]. A Cochrane review on community-based integrated packages to improve maternal and neonatal health found that community-based programming had a statistically significant positive impact on the initiation of breastfeeding within one hour of birth, with average RR = 1.94 [24].

Breastfeeding in the first hour of life is associated with prolonged duration of breastfeeding [25]. Commencement of breastfeeding immediately after birth ensures that the newborn receives the “first milk” (colostrum), the baby’s first immunization from diseases, which impacts the child’s good nutrition status [15]. Systematic reviews have highlighted the potential of early initiation of breastfeeding in preventing child undernutrition [25,26].

Use of any family planning method by mothers was also significantly associated with the child’s optimal nutrition status. A similar study in Ethiopia [18] showed that a child whose mother was not using family planning was about 2.5 times more likely to be stunted as compared to those who used family planning (AOR = 2.54 95% CI. 1.12–5.77). Even though birth interval was not included in this study an independent variable, family planning was used as an indication for birth spacing. This indicated that children born from mothers who had been using birth spacing were less likely to be affected by stunting.

Moreover, when family planning is not used to optimally space births, mothers are unlikely to provide adequate feeding practices to their children during the first two years of life. This puts the children at risk for undernutrition, subsequent illness and death [27]. In addition, if a mother gets pregnant soon after giving birth, she may prematurely wean the older infant off the breast. For instance, a study that explored reasons why mothers weaned their infants after one year of breastfeeding in Guinea-Bissau reported that 19% of mothers reported weaned due to a new pregnancy [27]. This implies that when births are too closely spaced, there is not enough time for a mother’s nutrient reserves to be replenished [28]. Thus, mothers would be overstrained to adequately satisfy the nutritional needs of both the breastfeeding baby and the newly conceived fetus. In addition, proper complementary feeding is also likely to be sub-optimal for poorly spaced births due to time, energy and other resource constraints [27].

On the other hand, more of the cases (84.0%) suffered an illness in the past two weeks prior to the study than the controls (67.0%), and the difference was statistically significant. This finding was in line with study findings reported from Ghana [28], where children that suffered no illness in the last 2 weeks were significantly more well nourished than those that reported to have suffered illness in the previous two weeks. Thus, disease/illness (for example, diarrhea) is a key immediate cause of malnutrition because it increases the body’s energy and nutrient expenditure and loss, hence depleting the body’s nutrient reserves and causing malnutrition [21].

Hand washing with soap was also an independent statistically significant practice associated with a child’s good nutrition status. For instance, the odds of a child being well nourished among caretakers who used soap for hand washing was 3.6 times the odds among caretakers who did not use soap. This finding agrees with several systematic reviews that have highlighted the potential of hand washing with soap to reduce diarrhea up to 48% [29]. Specifically, worms can be transferred through the fecal–oral route, which would be disrupted by good hand washing practices. For example, a cross-sectional survey in rural Andhra Pradesh India reported not using soap for hand washing as one of the strongest predictors of young child stunting [30].

A similar finding in India [20] reported that use of a hand washing agent (soap/ash/mud) compared with water alone was strongly and positively associated with normal nutrition status (HAZ) (β  =  0.317, 95% CI. 0.106–0.528, *p*  =  0.001). On the other hand, the World Health Organization (WHO) estimates that 50% of cases of child undernutrition are due to recurrent diarrhea and intestinal infections caused by poor sanitation and hygiene conditions [1]. Further, it is estimated that hand washing with soap can avert loss of nutrients through diarrhea and reduce stunting by up to 15% in children under five years of age, giving them a better chance of maintaining good nutrition status and growing up to thrive [21].

In this study, the number of under-fives in the household was statistically significantly associated with the optimal nutrition status of the children at multivariable analysis. Specifically, having less than five children aged below five years in a household was shown to be a significant predictor for good nutrition status even after adjusting for other confounders at multivariable analysis. Similar findings were reported in Ethiopia [18], Pakistan [31] and Malaysia [32]. In Ethiopia, children from households with less than three under-five children were about 4.5 times less likely to be undernourished (underweight) as compared to their counterparts from households with three or more children under-five (AOR = 4.52; 95% CI. 1.01–20.33) [18].

In Malaysia, households with four children and above (AOR: 5.86, 95% CI. 1.96–17.55) were at higher odds of having malnourished children as compared to households with less than four children. Another study conducted in an urban slum of India showed that the prevalence of underweight was significantly higher in households with more than three siblings. The households with no siblings had the lowest proportion of underweight children (12.8%), which was higher in households with one to two siblings (30.6%) and highest (51.7%) in those with more than three siblings [33]. The reduced number of children in families could have placed less burden on the household resources, particularly on finances and food; it might have increased the time and quality of care received by the children.

In this study, it was found that the maternal education was not significantly associated with children’s nutritional status at multivariable analysis, which is inconsistent with case control studies conducted in Nepal [12], India [19] and Ethiopia [34,35]. This discrepancy might have occurred because majority of the mothers involved in the study were illiterate.

This study was carried out to identify practices of mothers/caregivers associated with optimal nutrition status of their children aged 6–24 months in Gulu district, Northern Uganda. In this study, breastfeeding in the first hour of child’s life, child spacing through use of family planning methods, having a smaller number of children under five in the same household and hand washing with soap were practices significantly and independently associated with a child’s good nutrition status.

Key limitations of this study include that findings may be subject to social desirability bias, particularly because participants may have over-reported behaviors/practices known to be good or under-reported behaviors/practices known to be unfavorable in their society. This bias was reduced by asking neutral questions during interviews. Recall bias may also have affected the research findings; the mothers/caretakers of the cases may have had a better memory of potential risk factors for their children’s nutrition status than the mothers/caretakers of the controls. Considering a lower age group of 6–24-month-old children rather than 6–59 months old helped reduce this bias. The outcome of interest was generalized as nutrition status and not disaggregated into its components of stunting, underweight and wasting. The independent factors are therefore not specific to the nutrition status indices. The study sample was drawn from purposively selected communities in one district of Northern Uganda, which limits the generalizability of our findings.

## 5. Conclusions

The study results show that multi-disciplinary/multi-sectoral factors are associated with good nutrition status among children aged 6–24 months in the indigenous communities of Gulu district, Northern Uganda. The factors associated with the children’s normal nutrition status were: breastfeeding in the first hour of child’s life, use of family planning methods, having a smaller number of under-fives in the household and hand washing with soap. Therefore, early initiation of breastfeeding was the only feeding practice associated with normal nutrition status of children aged 6–24 months in Gulu district. Furthermore, having no history of illness in the past two weeks, hand washing with soap and use of family planning were the underlying health practices significantly associated with good nutrition status. In addition, having fewer under-five children in the household was the only social factor associated with the good nutrition status of children aged 6–24 months. Therefore, the study’s findings show that good nutrition status of children aged 6–24 months is an outcome of a complex of multi-disciplinary underlying and basic factors.

Although breastfeeding at least eight times a day was not found to be significantly associated with optimal nutrition status in this study, it is biologically plausible that frequent breastfeeding is a critical practice that needs to be encouraged among breastfeeding mothers. Further research is needed to determine the predictors of specific nutrition status indices that are disaggregated such as stunting, underweight and wasting.

## Figures and Tables

**Table 1:** Characteristics of study participants.

Variable	Cases (%) *n* = 100	Controls (%) *n* = 200	Total (%) *n* = 300
**Nutrition status**			
Male	57 (57.0)	91 (45.5)	148 (49.3)
Female	43 (43.0)	109 (54.5)	152 (50.7)
**Relationship of caretaker to child**			
Mother	88 (88.0)	187 (93.5)	275 (91.7)
Other (father, sister or grandparent)	12 (12.0)	13 (6.5)	25 (8.3)
**Education level of caretaker**			
No formal education	18 (18.0)	17 (8.5)	35 (11.7)
Primary	64 (64.0)	137 (68.5)	201 (67.0)
Secondary level or higher	18 (18.0)	46 (23.0)	64 (21.3)
**Marital status of caretaker**			
Married	28 (28.0)	94 (47.0)	122 (40.7) *
Married, polygamous	39 (39.0)	42 (21.0)	81 (27.0)
Cohabiting	18 (18.0)	36 (18.0)	54 (18.0)
Not married (single or separated)	15 (15.0)	28 (14.0)	43 (14.3)
**Age of caretaker**			
=<19 years	11 (11.2)	28 (14.0)	39 (13.0) *
20–34 years	65 (65.0)	147 (73.5)	212 (70.7)
>=35 years	24 (24.0)	25 (12.5)	49 (16.3)
**Family size**			
Average	**6**	**5**	**5**
=<5 individuals	50 (50.0)	132 (66.0)	182 (66.0) *
>5 individuals	50 (50.0)	68 (34.0)	118 (39.3)
**Number of U5s living in HH**			
0–2	76 (76.0)	185 (92.5)	261(87.0) *
3–7	24 (24.0)	15 (7.5)	39 (13.0)
**Source of income**	12 (12.0)	27 (13.5)	39 (13.0)
Not employed	79 (79.0)	149 (74.5)	228 (76.0)
Farming Business/trade	8 (8.0)	21 (10.5)	29 (9.7)
Formal employment	1 (1.0)	3 (1.5)	4 (1.3)
**Family earnings per day**			
1 USD or less/day	94 (94.0)	166 (83.0)	260 (86.7)
More than 1 USD/day	6 (6.0)	34 (17.0)	40 (13.3) *
**Participation in community events**			
Yes	51 (51.0)	119 (59.5)	170 (56.7)
No	49 (49.0)	81 (40.5)	130 (43.3)

Pearson chi-square test; significant at *p* < 0.05. U5s = children under-five, USD = United States Dollars, community events = teachings, sports and SACCOs. (* *p* < 0.05).

**Table 2:** Comparison of child-feeding practices of cases and controls.

Variable	Cases (%) *n* = 100	Controls (%) *n* = 200	*p*-Value
**Child’s history of breastfeeding**			
Ever breastfed	97 (97.0)	197 (98.5)	0.382
Not breastfed	3 (3.0)	3 (1.5)	
**Initiation of breastfeeding**			
<1 h	77 (79.4)	177 (89.9)	0.014
After 1 h	20 (20.6)	20 (10.1)	
**Introduction of CF ^a^**			
Before 6 months	57 (57.0)	90 (45.0)	
At 6 months	40 (40.0)	96 (48.0)	0.092
After 6 months	3 (3.0)	14 (7.0)	
**Frequency of breastfeeding**			
<8 times/day	53 (54.6)	67 (34.0)	0.001
>=8 times/day	44 (45.4)	130 (66.0)	
**Mode of feeding**			
Using hand	94 (94.0)	181 (90.5)	0.301
Others ^b^	6 (6.0)	19 (9.5)	
**Infant feeding during sickness**			
Feed more frequently	40 (40.0)	84 (42.0)	
Give more amounts of food	19 (19.0)	36 (18.0)	0.929
Give more fluids	32 (32.0)	61 (30.5)	
Combination of the above	9 (9.0)	19 (9.5)	
**Duration of breastfeeding**			
<12 months	2 (2.3)	1 (0.6)	0.258
>=12 months	84 (97.7)	171 (99.4)	

Chi-square test; significant with *p* < 0.05 at 95% CI. ^a^ CF = complementary feeding; ^b^ Others = cup, spoon or bottle-feeding.

**Table 3:** Comparison of caring practices of caretakers of cases and controls.

Variable	Cases (%) *n* = 100	Controls (%) *n* = 200	*p*-Value
**Handwashing with soap**			
Yes	73 (73.0)	180 (90.0)	0.000
No	27 (27.0)	20 (10.0)	
**Caretaker’s knowledge of WASH**			
Yes	59 (59.0)	130 (65.0)	0.310
No	41 (41.0)	70 (35.0)	
**Disciplining a child**			
Talk to child	57 (57.0)	117 (58.5)	0.804
Punish the child	43 (43.0)	83 (41.5)	
**Knowledge of basic needs of a child**			
Food	76 (76.0)	164 (82.0)	0.221
Other basic needs (see narrative)	24 (24.0)	36 (18.0)	
**Spouse/partner support**			
Ensuring food is available	70 (70.0)	166 (83.0)	0.010
Others ^a^	30 (30.0)	34 (17.0)	

Significant at *p* < 0.05 at 95% CI; WASH = water, sanitation and hygiene; ^a^ Other basic needs = sleep, clothing, shelter, education or immunization.

**Table 4:** Comparison of health-seeking practices of caretakers of cases and controls.

Variable	Cases (%) *n* = 100	Controls (%) *n* = 200	*p*-Value
**ANC ^a^ attendance**			
Yes No	82 (82.0)	185 (92.5)	0.006
	18 (18.0)	15 (7.5)	
**ANC visits**			
<4 visits	35 (35.0)	32 (16.0)	0.000
>=4 visits	65 (65.0)	168 (84.0)	
**Child immunized**			
Yes	94 (94.0)	197 (98.5)	0.031
No	6 (6.0)	3 (1.5)	
**Common child illnesses**			
Fever/malaria	77 (77.0)	162 (81.4)	0.369
^b^ Other illnesses	23 (23.0)	37 (18.6)	
**Management of child illnesses**			
Took child to health facility	80 (80.0)	178 (89.0)	0.078
Gave child local herbs	19 (19.0)	20 (10.0)	
Prayed for child	1 (1.0)	1 (0.5)	
Do not know	0	1 (0.5)	
**History of illness in the last 2 weeks**			
Had illness	84 (84.0)	134 (67.0)	0.002
No illness	16 (16.0)	66 (33.0)	
**Use of any FP method**			
Yes	37 (37.0)	109 (54.5)	0.004
No	63 (63.0)	91 (45.5)	
**Parity**			
>=4 pregnancies	48 (48.0)	50 (25.0)	0.000
<4 pregnancies	52 (52.0)	150 (75.0)	
**Use of mosquito net**			
Yes	68 (68.0)	161 (80.5)	0.016
No	32 (32.0)	39 (19.5)	

Significant at *p* < 0.05 at 95% CI; **^a^** ANC = antenatal care; **^b^** diarrhea, cough and flu; FP = family planning.

**Table 5:** Feeding, caring and health-seeking practices associated with optimal nutrition status (Case = 0, Control = 1).

Variable	Cases, *N* (%)	Controls, *N* (%)	Crude OR (95% CI)
**Child’s history of breastfeeding**			
Ever breastfed	3 (3.0)	3 (1.5)	1
Not breastfed	97 (97.0)	197 (98.5)	2.03 (0.40, 10.25)
**Initiation of breastfeeding**			
After 1 h	20 (20.6)	20 (10.1)	1
<1 h	77 (79.4)	177 (89.9)	2.29 (1.16, 4.49)
**Frequency of breastfeeding**			
<8 times/day	53 (54.6)	67 (34.0)	1
>=8 times/day	44 (45.4)	130 (66.0)	2.34 (1.42, 3.84)
**Hand washing with soap**			
No	27 (27.0)	20 (10.0)	1
Yes	73 (73.0)	180 (90.0)	3.34 (1.78, 6.31)
**ANC attendance**			
No	18 (18.0)	15 (7.5)	1
Yes	82 (82.0)	185 (92.5)	2.71 (1.30, 5.63)
**ANC visits**			
<4 visits	35 (35.0)	32 (16.0)	1
>=4 visits	65 (65.0)	168 (84.0)	2.83 (1.33, 4.45)
**History of illness in the last 2 weeks**			
Had illness	84 (84.0)	134 (67.0)	1
No illness	16 (16.0)	66 (33.0)	2.59 (1.40, 4.76)
**Use of any FP method**			
No	63 (63.0)	91 (45.5)	1
Yes	37 (37.0)	109 (54.5)	2.04 (1.24, 3.34)
**Parity**			
=<4 pregnancies	52 (52.0)	150 (75.0)	1
>4 pregnancies	48 (48.0)	50 (25.0)	0.36 (0.22, 0.60)
**Use of mosquito nets**			
No	32 (32.0)	39 (19.5)	1
Yes	68 (68.0)	161 (80.5)	1.94 (1.12, 3.36)

Significant with *p* < 0.05 at 95% CI; FP = family planning.

**Table 6:** Social factors associated with child optimal nutrition status (Case = 0, Control = 1).

Variable	Cases, *N* (%)	Controls, *N* (%)	Crude OR (95% CI)
**Education level of caretaker**			
No formal education	18 (18.0)	17 (8.5)	1
Primary	64 (64.0)	137 (68.5)	2.27 (1.09, 4.69)
Secondary level or higher	18 (18.0)	46 (23.0)	2.71 (1.15, 6.38)
**Marital status of caretaker**			
Married, monogamous	28 (28.0)	94 (47.0)	1
Married, polygamous	39 (39.0)	42 (21.0)	0.28 (0.15, 0.53)
Cohabiting	18 (18.0)	36 (18.0)	0.52 (0.25, 1.08)
Not married	15 (15.0)	28 (14.0)	0.48 (0.22, 1.06)
**Number of U5s ^a^ in HH ^b^**			
0–2	76 (76.0)	185 (92.5)	1
3–7	24 (24.0)	15 (7.5)	0.39 (0.19, 0.82)
**Family earnings per day**			
More than 1 USD/day	6 (6.0)	34 (17.0)	2.93 (1.18, 7.27)
1 USD or less/day	94 (94.0)	166 (83.0)	1
**Age of caretaker**			
=<19 years	11 (11.2)	28 (14.0)	1
20–34 years	65 (65.0)	147 (73.5)	0.89 (0.42, 1.89)
>=35 years	24 (24.0)	25 (12.5)	0.41 (0.17, 1.00)
**Family size**			
=<5 individuals	50 (50.0)	132 (66.0)	1
>5 individuals	50 (50.0)	68 (34.0)	0.52 (0.32, 0.84)
**Participation in community events**			
Yes	51 (51.0)	119 (59.5)	1.4 (0.87, 2.29)
No	49 (49.0)	81 (40.5)	1

^a^ U5s = under-fives; ^b^ HH = household.

**Table 7:** Factors associated with optimal nutrition status (multivariable analysis, case = 0, control = 1).

Variable	Cases, *N* (%)	Controls, *N* (%)	Crude OR (95% CI)	Adjusted OR (95% CI)
**Initiation of breastfeeding**				
<1 h	77 (79.4)	177 (89.9)	2.29 (1.16, 4.49)	3.31 (1.52, 7.23)
After 1 h	20 (20.6)	20 (10.1)	1	1
**Use of any ^a^ FP method**				
Yes	37 (37.0)	109 (54.5)	2.04 (1.24, 3.34)	2.21 (1.25, 3.90)
No	63 (63.0)	91 (45.5)	1	1
**Number of ^b^ U5s in ^c^ HH**				
3–7	24 (24.0)	15 (7.5)	0.39 (0.19, 0.82)	0.31 (0.13, 0.73)
0–2	76 (76.0)	185 (92.5)	1	1
**Hand washing with soap**				
Yes	73 (73.0)	180 (90.0)	3.34 (1.78, 6.31)	3.63 (1.76, 7.49)
No	27 (27.0)	20 (10.0)	1	1
**Education level of caretaker**				
No formal education	18 (18.0)	17 (8.5)	1	1
Primary	64 (64.0)	137 (68.5)	2.27 (1.09, 4.69)	1.23 (0.53, 2.85)
Secondary level or higher	18 (18.0)	46 (23.0)	2.71 (1.15, 6.38)	1.55 (0.59, 4.07)
**Family earnings per day**				
More than 1 USD/day	6 (6.0)	34 (17.0)	2.93 (1.18, 7.27)	2.13 (0.83, 5.44)
1 USD or less/day	94 (94.0)	166 (83.0)	1	1
**Use of mosquito nets**				
No	32 (32.0)	39 (19.5)	1	1
Yes	68 (68.0)	161 (80.5)	1.94 (1.12, 3.36)	0.93 (0.48, 1.81)
**History of illness in the last 2 weeks**				
Had illness	84 (84.0)	134 (67.0)	1	1
No illness	16 (16.0)	66 (33.0)	2.59 (1.40, 4.76)	1.95 (1.01, 3.77)
**Frequency of breastfeeding**				
<8 times/day	53 (54.6)	67 (34.0)	1	1
>=8 times/day	44 (45.4)	130 (66.0)	2.34 (1.42, 3.84)	1.72 (0.90, 3.01)

^a^ Family planning; ^b^ under-fives; ^c^ household.

## Data Availability

The data presented in this study are available on request from the corresponding author on reasonable request.

## References

[1] WHO Infant and Young Child Feeding 2021. https://www.who.int/news-room/fact-sheets/detail/infant-and-young-child-feeding.

[2] Khan S., Zaheer S., Safdar N.F. (2019). Determinants of stunting, underweight and wasting among children <5 years of age: Evidence from 2012–2013 Pakistan demographic and health survey. BMC Public Health.

[3] Nankumbi J., Muliira J.K. (2015). Barriers to Infant and Child-feeding Practices: A Qualitative Study of Primary Caregivers in Rural Uganda. J. Health Popul. Nutr..

[4] UBOS (2018). Uganda Demographic and Health Survey 2016.

[5] MoH (2015). A Guide for Integrated Community-Based Management of Malnutrition Using Positive Deviance/Hearth.

[6] Bullen P.A.B. (2011). The positive deviance/hearth approach to reducing child malnutrition: Systematic review. Trop. Med. Int. Heal..

[7] Picho E.O., Namubiru M., Ngaka W. (2018). The Effect of ICT on Households’ Food Security in Uganda. Evidence from Acholi Sub Region in Northern Uganda. chrome-extension://efaidnbmnnnibpcajpcglclefindmkaj/viewer.html?pdfurl=http%3A%2F%2Fdir.muni.ac.ug%2Fbitstream%2Fhandle%2F20.500.12260%2F300%2FPicho-Article-2018_2.pdf%3Fsequence%3D1%26isAllowed%3Dy&clen=151254.

[8] Fleiss J.L., Levin B., Paik M.C. (2013). Statistical Methods for Rates and Proportions.

[9] Lewallen S., Courtright P. (1998). Epidemiology in practice: Case-control studies. Community Eye Health.

[10] Perneger T.V., Courvoisier D.S., Hudelson P.M., Gayet-Ageron A. (2015). Sample size for pre-tests of questionnaires. Qual. Life Res..

[11] Forsido S.F., Tsegaye N.K., Tamiru D., Belachew T., Hensel O. (2021). Undernutrition and associated factors among children under 2 years of age in Jimma Zone, Southwest Ethiopia. J. Public Health.

[12] Adhikari N., Acharya K., Upadhya D.P., Pathak S., Pokharel S., Pradhan P.M.S. (2021). Infant and young child feeding practices and its associated factors among mothers of under two years children in a western hilly region of Nepal. PLoS ONE.

[13] Bukusuba J., Kaaya A.N., Atukwase A. (2017). Predictors of Stunting in Children Aged 6 to 59 Months: A Case–Control Study in Southwest Uganda. Food Nutr. Bull..

[14] Anino O.C., Were G.M., Khamasi J.W. (2015). Impact evaluation of positive deviance hearth in Migori county, Kenya. Afr. J. Food Agric. Nutr. Dev..

[15] WHO (2013). WHO Guidelines Approved by the Guidelines Review Committee, in Essential Nutrition Actions: Improving Maternal, Newborn, Infant and Young Child Health and Nutrition.

[16] Dhami M.V., Ogbo F.A., Diallo T.M., Olusanya B.O., Goson P.C., Agho K.E., on behalf of the Global Maternal and Child Health Research Collaboration (GloMACH) (2021). Infant and Young Child Feeding Practices among Adolescent Mothers and Associated Factors in India. Nutrients.

[17] Turcan L., Bene T. (2017). A Review of Policies for Improving Human Nutrition in Uganda and the Use of Evidence for Making Policy.

[18] Tadele T.T., Gebremedhin C.C., Markos M.U., Fitsum E.L. (2022). Stunting and associated factors among 6–23 month old children in drought vulnerable kebeles of Demba Gofa district, southern Ethiopia. BMC Nutr..

[19] Mishra K., Kumar P., Basu S., Rai K., Aneja S. (2013). Risk Factors for Severe Acute Malnutrition in Children below 5 y of Age in India: A Case-Control Study. Indian J. Pediatr..

[20] Saxton J., Rath S., Nair N., Gope R., Mahapatra R., Tripathy P., Prost A. (2016). Handwashing, sanitation and family planning practices are the strongest underlying determinants of child stunting in rural indigenous communities of Jharkhand and Odisha, Eastern India: A cross-sectional study. Matern. Child Nutr..

[21] Nutrition I. (2013). The Achievable Imperative for Global Progress New York.

[22] Oddy W.H. (2013). Breastfeeding in the first hour of life protects against neonatal mortality. J. Pediatr..

[23] Muchina E., Waithaka P. (2010). Relationship between breastfeeding practices and nutritional status of children aged 0–24 months in Nairobi, Kenya. Afr. J. Food Agric. Nutr. Dev..

[24] Lassi Z.S., Rind F., Irfan O., Hadi R., Das J.K., Bhutta Z.A. (2020). Impact of Infant and Young Child Feeding (IYCF) Nutrition Interventions on Breastfeeding Practices, Growth and Mortality in Low- and Middle-Income Countries: Systematic Review. Nutrients.

[25] Thukral A., Sankar M.J., Agarwal R., Gupta N., Deorari A.K., Paul V.K. (2012). Early Skin-to-Skin Contact and Breast-Feeding Behavior in Term Neonates: A Randomized Controlled Trial. Neonatology.

[26] Sharma I.K., Byrne A. (2016). Early initiation of breastfeeding: A systematic literature review of factors and barriers in South Asia. Int. Breastfeed. J..

[27] Naik R., Smith R. (2015). Impacts of Family Planning on Nutrition.

[28] Mahama A.M., Anaman K.A., Osei-Akoto I. (2014). Factors influencing householders' access to improved water in low-income urban areas of Accra, Ghana. J. Water Health.

[29] Cairncross S., Hunt C., Boisson S., Bostoen K., Curtis V., Fung I.C.-H., Schmidt W.-P. (2010). Water, sanitation and hygiene for the prevention of diarrhoea. Int. J. Epidemiol..

[30] Bansal S.C., Odedra R., Talati K., Morgaonkar V.A., Shinde M., Nimbalkar S.M. (2021). Infant and young child feeding (IYCF) practices and their determinants in two Urban districts of India. J. Fam. Med. Prim. Care.

[31] Khattak M.K., Ali S. (2010). Malnutrition and associated risk factors in pre-school children (2–5 years) in District Swabi (NWFP)-Pakistan. J. Med Sci..

[32] Wong H.J., Moy F.M., Nair S. (2014). Risk factors of malnutrition among preschool children in Terengganu, Malaysia: A case control study. BMC Public Health.

[33] Sengupta P., Philip N., Benjamin A. (2010). Epidemiological correlates of under-nutrition in under-5 years children in an urban slum of Ludhiana. Health Popul. Perspect. Issues.

[34] van Eijsden M., Smits L.J., van der Wal M.F., Bonsel G.J. (2008). Association between short interpregnancy intervals and term birth weight: The role of folate depletion. Am. J. Clin. Nutr..

[35] Mekonnen M., Kinati T., Bekele K., Tesfa B., Hailu D., Jemal K. (2021). Infant and young child feeding practice among mothers of children age 6 to 23 months in Debrelibanos district, North Showa zone, Oromia region, Ethiopia. PLoS ONE.

